# A special issue dedicated to the memory of Jonathan R. Warner (1936-2019), a pioneer of ribosome biology

**DOI:** 10.1080/15476286.2023.2221944

**Published:** 2023-06-07

**Authors:** Marlene Oeffinger

**Affiliations:** Institut de Recherches Cliniques de Montréal, Canada; Département de Biochimie, Faculté de Médecine, Université de Montréal, QC, Canada; Faculty of Medicine, Division of Experimental Medicine, McGill University, Montréal, QC, Canada

I first met Jon Warner, when I interviewed for a postdoctoral position in his lab in November of 2002. I had been conducting my graduate work on ribosome biogenesis in David Tollervey’s lab in Edinburgh, and not yet ready to let go of the fascinating conundrum that is ribosome maturation, I was considering continuing on in the field. Naturally, one of the places I applied to was Jonathan Warner’s group at the Albert Einstein College of Medicine in New York City and I was invited for an interview, which back then still took place in person.

Jonathan Warner (1936-2019) was an American molecular biologist and pioneer in both the fields of translation and ribosome biogenesis. Among his many key discoveries are those of polyribosomes in 1963 and correlating the number of tRNAs, nascent polypeptide chains to that of ribosomes within polysomes and hence demonstrating their activity in translation a year later. From the 1970s onwards all the way into the 2010s, his research focus expanded from translation regulation to ribosome maturation. His research led to the ‘discovery’ of extraribosomal proteins on nuclear 80S pre-ribosomal particles (now known as ribosome maturation factors), defining the coordination of ribosomal protein and rRNA synthesis as well as the link between cell starvation and shutdown of rRNA transcription, or ‘stringent response’, among numerous other things, including ribosomal protein synthesis and mechanisms of rDNA transcription in *S. cerevisiae*. In the last decade, his interest was drawn to the evolution of ribosomal proteins.

While it was his work on ribosomes that drew me to apply to Jon’s lab, what struck me about our initial meeting was not only encountering someone as fascinated by ribosomes as myself, but more so his caring and his earnest interest in others. Over the years and many meetings with Jon, I would always appreciate his passion for ribosomes and science as a whole, the avid discussions and candid advice – and beyond that the compassion he had in particular for the younger generation of scientists that attempted to forge a career in science. While I did not join his lab at the time (based on his advice, in fact, to step ‘sideways’, outside of the field, for my postdoctoral stage), I have always considered Jon a mentor since that meeting. I was lucky to have had great conversations with him over the years, be it on ribosomes, RNA biology, or the next attempted step in my career path, and I have always tried to follow his example to approach science with passion and compassion.

When the then Editor in Chief of *RNA Biology*, Renée Schroeder, invited me as guest editor to put together a Special Issue on the Ribosome Life cycle, my aim became to mirror in some form the kind of conversations that Jon Warner brought to the field and provide a candid perspective of our current state of knowledge on ribosome biogenesis and translation and the challenges ahead. I wish all the readers of this special issue an interesting time while getting acquainted with the ribosome and hope they will be able to appreciate the fascination this multifaceted macromolecule held for Jonathan Warner – as it still does for many of us in the field.

